# Placental Pathophysiology and Developmental Programming of Adult Cardiometabolic Risk: A Narrative Review of Pregnancy Exposures

**DOI:** 10.3390/pathophysiology33030067

**Published:** 2026-09-08

**Authors:** Eleftherios Panteris, Ioanna Kakatsaki, Zoi Koukou, Charalambos Kolvatzis, Styliani Papanikolaou, Eleftheria Hatzidaki

**Affiliations:** 1 Department of Neonatology/Neonatal Intensive Care Unit, University Hospital of Heraklion, School of Medicine, University of Crete, 70013 Heraklion, Greece; eleftherios.panteris@gmail.com (E.P.); joanne_k1991@hotmail.com (I.K.); stylpapa12@gmail.com (S.P.); 2 School of Health Sciences, International Hellenic University (IHU), Sindos, 57400 Thessaloniki, Greece; koukou.zoi@ihu.gr; 3 Third Department of Obstetrics and Gynecology, School of Medicine, Faculty of Health Sciences, Aristotle University of Thessaloniki, 54124 Thessaloniki, Greece; charis_kolv@hotmail.com

**Keywords:** DOHaD, placenta, fetal programming, pregnancy complications, fetal growth restriction, preterm birth, epigenetics, cardiometabolic disease

## Abstract

Non-communicable diseases remain the dominant cause of adult morbidity and mortality, yet cardiometabolic, vascular and renal risk may originate before birth. This structured narrative review critically evaluates human evidence linking pregnancy-related exposures, placental pathophysiology and later offspring health within a maternal–placental–fetal–life-course framework. Evidence published from 2020 to 2026 was emphasised, while seminal earlier cohorts were retained when they provided uniquely long follow-up or direct placental measurements. The most consistent associations concern maternal obesity, gestational diabetes, excessive gestational weight gain and hypertensive disorders, which are linked with higher offspring blood pressure, greater adiposity and adverse glucose–insulin profiles. Human placental studies implicate vascular malperfusion, altered nutrient transport, inflammatory and oxidative signalling, endocrine function and epigenetic regulation. Few studies, however, have measured the prenatal exposure, a specific placental phenotype and a long-term offspring outcome within the same longitudinal design; most proposed placental pathways are supported by convergent evidence or biological plausibility rather than demonstrated mediation. Preterm birth and fetal growth restriction are clinically observable, etiologically heterogeneous sentinel phenotypes, not obligatory mediators. Placental and offspring epigenetic signatures, telomere biology and mitochondrial function represent distinct domains of biological embedding; their causal and prognostic significance remains uncertain. Longitudinal studies integrating maternal exposures, placental histopathology and molecular phenotypes, fetal organ development, childhood cardiometabolic trajectories and adult clinical outcomes are needed to determine whether, and under what circumstances, the placenta plays an intermediary role rather than merely serving as a marker of an adverse pregnancy environment.

## 1. Introduction

The global burden of chronic non-communicable diseases (NCDs) is substantial and continues to rise, accounting for nearly two-thirds of disability-adjusted life years and deaths worldwide [1]. Cardiovascular disease (CVD), neoplasms, chronic respiratory diseases, and diabetes are the leading contributors, with cardiovascular disease responsible for the largest proportion of NCD-related mortality. In addition to mortality, NCDs impose long-term disability, reduced productivity, and considerable economic strain on individuals, families, and health systems [2,3]. Although NCDs are typically diagnosed in mid- to late adulthood, growing evidence indicates that they originate much earlier, beginning in fetal life and evolving across the lifespan. The developmental origins of health and disease (DOHaD) framework builds on this concept, proposing that environmental exposures during critical developmental periods have lasting effects on physiology and influence later cardiometabolic risk [4,5,6].

The DOHaD framework integrates evolutionary, physiological, and molecular perspectives to explain how early-life environmental exposures shape long-term health. The concept emerged from epidemiological findings demonstrating that low birth weight and early nutritional adversity are associated with increased risk of adult CVD and type 2 diabetes [7,8]. Building on these findings, the framework proposes that exposures during critical developmental windows—particularly during the first 1000 days of life—induce persistent changes in organ structure, metabolic pathways, and regulatory systems. This period has emerged as a sensitive phase during which environmental and biological exposures may shape long-term telomere dynamics and ageing-related trajectories [9]. Epigenetic mechanisms, including DNA methylation and histone modification, are considered key mediators through which early-life conditions influence gene expression and later disease susceptibility [10,11]. Evidence supporting the DOHaD paradigm has strengthened as large population-based cohorts with extended follow-up have matured, enabling direct linkage of perinatal characteristics—such as gestational age and fetal growth—with adult cardiometabolic and neurobehavioural outcomes [12,13]. These long-term data provide robust support for the concept that developmental processes contribute meaningfully to disease risk well beyond childhood and adolescence.

Pregnancy represents a critical developmental window during which fetal organs undergo rapid growth and differentiation within a tightly regulated intrauterine environment [14]. The placenta is a dynamic sensor and effector at the maternal–fetal interface: maternal metabolic, vascular, inflammatory and endocrine conditions can alter placental perfusion, transport, signalling and endocrine function, with consequences for fetal substrate exposure and organ development [15,16]. Maternal nutritional interventions may modify the risk of pregnancy outcomes linked to placental dysfunction, although the evidence varies in quality and does not establish an effect on long-term offspring disease [17]. Placental responses may contribute to developmental programming through structural, functional and epigenetic adaptation, but the placenta should not be assumed to mediate every association between a prenatal exposure and later disease. Direct fetal effects, maternal and fetal genetics, shared family factors and the postnatal environment may also shape the observed trajectory [18,19,20].

Accordingly, preterm birth and fetal growth restriction (FGR) should be regarded as clinically observable sentinel phenotypes that may reflect several maternal–placental–fetal pathways, rather than as simple intermediate steps. Preterm birth can arise through infection and inflammation, cervical or membrane pathways, maternal vascular disease, placental disorders, fetal disease or medically indicated delivery [21]; FGR is often associated with placental insufficiency but is not synonymous with it [22,23]. Both phenotypes are associated with later cardiometabolic risk, yet neither is a specific or obligatory mediator [23]. Early postnatal exposures, including breastfeeding, may further modify growth and cardiometabolic trajectories, although reported long-term effects are generally modest and should be interpreted alongside later behavioural and clinical risk factors [24].

The aim of this review is to consolidate and critically appraise human evidence linking pregnancy exposures, measured placental phenotypes, fetal development and sentinel birth phenotypes with cardiometabolic outcomes across childhood and adulthood. The review focuses on hypertension, adverse adiposity and dysglycaemia, while considering cardiovascular, renal and selected neuropsychiatric outcomes, where they clarify the broader life-course consequences. Particular attention is given to the strength of evidence supporting the proposed biological pathway: whether the exposure, placental phenotype and offspring outcome were assessed within the same cohort; whether successive links in the pathway are supported by separate human studies; or whether the proposed intermediary role of the placenta remains biologically plausible but untested.

**Figure 1:**
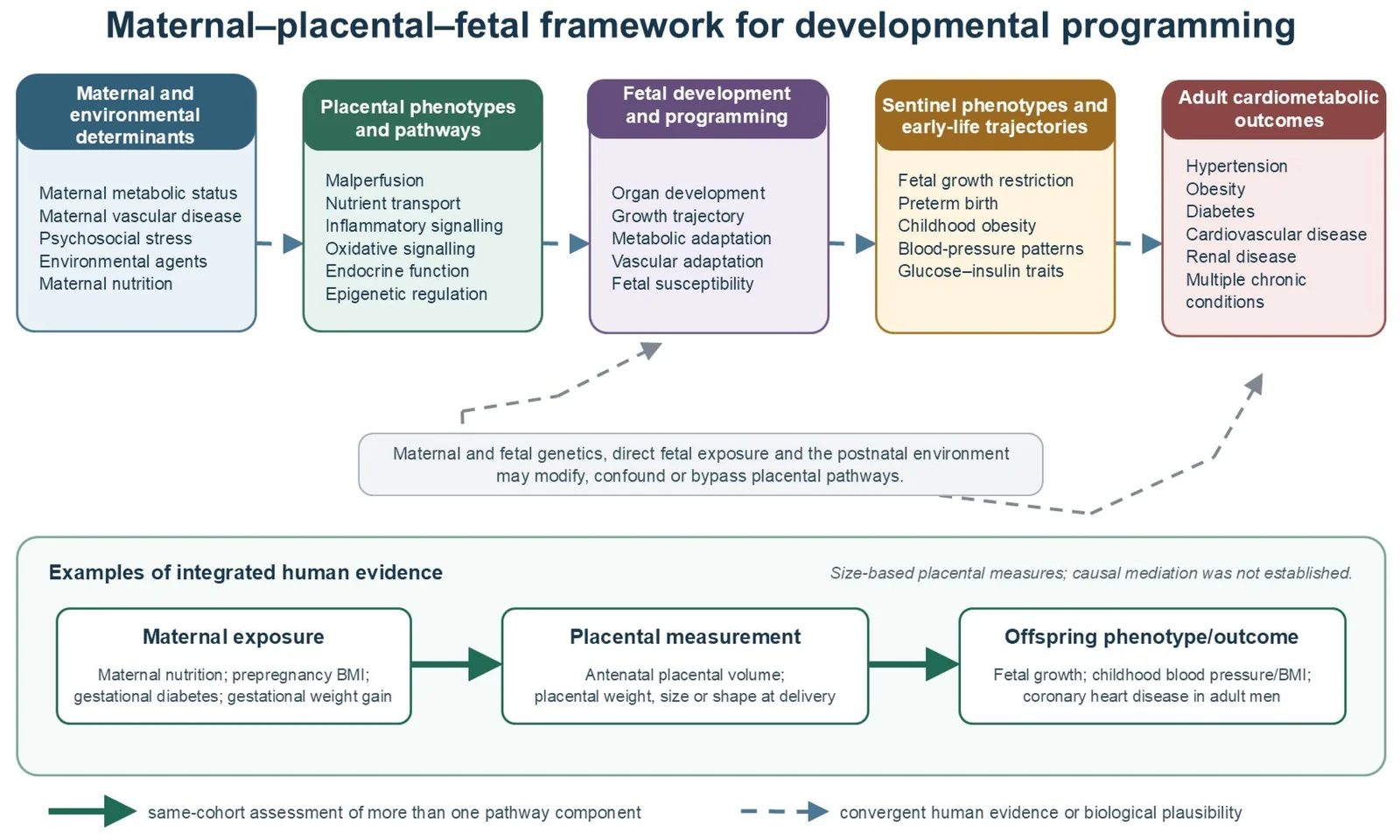
Maternal–placental–fetal framework for developmental programming. Upstream maternal and environmental exposures may alter placental perfusion, transport, inflammatory, oxidative, endocrine and molecular functions. Placental responses interact with fetal susceptibility and may influence organ development and clinically observable sentinel phenotypes, including fetal growth restriction and preterm birth. Childhood cardiometabolic trajectories and postnatal exposures further shape the risk of cardiometabolic disease in adulthood. Solid arrows denote examples in which more than one pathway component was assessed within the same human cohort; dashed arrows denote relationships supported by convergent evidence or biological plausibility. The diagram does not imply that the placenta is a universal mediator or that preterm birth or fetal growth restriction is an obligatory mediator.

The framework used throughout the review is shown in Figure 1. Upstream metabolic, vascular, psychosocial and environmental determinants are considered in relation to cross-cutting placental phenotypes, including vascular malperfusion, altered nutrient transport, inflammatory and oxidative signalling, endocrine function and epigenetic regulation. These placental responses may interact with fetal susceptibility and organ development, appear clinically as sentinel pregnancy or birth phenotypes, and contribute to childhood cardiometabolic trajectories and adult disease. The framework does not assume universal placental mediation; it distinguishes integrated human evidence from convergent associations and biological plausibility.

## 2. Methods

### 2.1. Design and Reporting Approach

This structured narrative review used iterative, section-specific searches to identify epidemiological, clinical, placental and molecular evidence. Searches were organised around predefined concept blocks and updated as each thematic section was developed. The review was not based on a single deduplicated screening set; a PRISMA flow diagram, formal risk-of-bias assessment and meta-analysis were not undertaken. This design was selected to permit critical integration of heterogeneous exposure domains, placental phenotypes, study designs and long-term offspring outcomes.

PubMed/MEDLINE was the primary biomedical database, supplemented by Scopus for environmental and exposure-science literature not consistently indexed in MEDLINE. Evidence published from 1 January 2020 to 9 August 2026 was prioritised for contemporary exposure assessment, placental biology, molecular biomarkers and systematic reviews. Seminal earlier epidemiological and experimental studies were included irrespective of publication year when they established the developmental origins or placental-programming paradigms, provided uniquely long follow-up, or included direct placental measurements. Reference lists of eligible reviews and landmark cohort papers were also hand-checked.

### 2.2. Eligibility Criteria

We included: (a) systematic reviews and meta-analyses; (b) population-based cohorts and register studies; (c) pregnancy studies measuring a defined maternal exposure together with a placental structural, vascular, endocrine, inflammatory, epigenetic or other molecular phenotype, even when long-term offspring follow-up was unavailable; (d) longitudinal offspring studies extending into childhood, adolescence or adulthood, including exposure–outcome studies without placental measurements; and (e) same-cohort studies assessing at least two stages of the proposed pathway. High-quality narrative reviews were retained when they provided clinically relevant integration or methodological guidance. Animal-only studies, reports limited to acute neonatal outcomes without relevant placental measurements, editorials without primary data or explicit review methods, and studies in which pregnancy exposure could not be separated meaningfully from postnatal exposure were excluded. Placental-only studies were treated as adjacent-link evidence and not as proof that a placental phenotype mediates a later offspring outcome. The framework therefore distinguishes integrated human evidence from convergent associations and biological plausibility, without assuming universal placental mediation.

Because the searches were iterative and section-specific, no single set of records identified, screened and excluded could be reconstructed without implying a systematic screening process that had not been undertaken. Formal certainty grading and study-level risk-of-bias scoring were not performed. Instead, interpretation considered study design, sample size, follow-up duration, exposure and outcome measurement, adjustment for confounding, consistency across studies and whether placental and offspring measures were obtained within the same cohort.

### 2.3. Framework for Evidence Interpretation

Evidence linking prenatal exposures, placental phenotypes and offspring outcomes was classified into four interpretative categories. Integrated longitudinal human evidence referred to studies in which the prenatal exposure, a placental phenotype or pathway and an offspring phenotype or outcome were measured within the same cohort. Convergent human evidence referred to adjacent links supported by separate human studies. Exposure–outcome association denoted epidemiological evidence without direct placental measurement, while biological plausibility denoted pathways supported by placental, cellular or experimental evidence without longitudinal confirmation in humans.

These categories were used to prevent statistical association, biological plausibility and demonstrated placental mediation from being treated as equivalent. They are interpretive descriptors rather than a formal evidence-grading system.

### 2.4. Search Strings (PubMed Examples)

The following PubMed strings were used as templates and adapted for each section by inserting domain-specific terms. Filters were applied to include studies involving humans and published in English; no restrictions were placed on country.

#### 2.4.1. Core Life-Course Filter

((pregnan* OR prenatal OR antenatal OR intrauterine OR “in utero” OR gestation* OR placent*) AND (“Developmental Origins” OR DOHaD OR “fetal programming” OR “life course” OR “early life”) AND (adult* OR adolescence OR “young adult” OR “midlife” OR “long-term”))

#### 2.4.2. Maternal Metabolic/Vascular Health

((pregnan* OR prenatal OR antenatal) AND (obes* OR “body mass index” OR “gestational diabetes” OR hyperglyc* OR “weight gain” OR preeclampsia OR “hypertensive disorders”) AND (offspring OR child* OR adolescent* OR adult*) AND (blood pressure OR hypertension OR cardiometabolic OR diabetes OR insulin OR lipid* OR “cardiovascular” OR “kidney” OR “chronic kidney disease”))

#### 2.4.3. Psychosocial Stress/Mental Health

((pregnan* OR prenatal OR antenatal) AND (stress OR bereavement OR depression OR anxiety OR antidepressant*) AND (offspring OR child* OR adolescent* OR adult*) AND (“cardiovascular” OR hypertension OR diabetes OR “mental health” OR depression OR anxiety OR multimorbid*))

#### 2.4.4. Environment/Chemicals/Diet/Built Environment

((pregnan* OR prenatal OR antenatal) AND (“air pollution” OR PM2.5 OR PM10 OR NO_2_ OR metals OR lead OR cadmium OR mercury OR PFAS OR “perfluoro” OR bisphenol* OR phthalate* OR “ultra-processed” OR diet OR greenspace) AND (offspring OR child* OR adolescent* OR adult*) AND (blood pressure OR hypertension OR obesity OR adiposity OR diabetes OR “cardiometabolic” OR “vascular”))

#### 2.4.5. Ageing Biomarkers

((pregnan* OR prenatal OR antenatal) AND (telomere* OR “telomere length” OR “epigenetic age” OR “epigenetic age acceleration” OR “DNA methylation” OR mtDNA OR mitochondrial) AND (offspring OR newborn OR infant OR child* OR adolescent* OR adult*))

In Scopus, the same concept blocks were used with TITLE-ABS-KEY fields. An example template was: TITLE-ABS-KEY((pregnan* OR prenatal OR antenatal OR intrauterine OR “in utero”) AND (air pollution OR PM2.5 OR NO_2_ OR PFAS OR bisphenol* OR phthalate* OR metals) AND (offspring OR adolescent* OR adult*) AND (hypertension OR blood pressure OR cardiometabolic OR diabetes OR stroke OR heart failure)).

## 3. Maternal Metabolic and Vascular Disorders: Exposure–Placenta–Offspring Pathways

Maternal metabolic and vascular health during pregnancy can alter placental perfusion, nutrient transport, inflammatory signalling and endocrine function, with potential consequences for fetal growth and cardiometabolic development [25,26]. The most consistent exposure–outcome associations concern maternal obesity, excessive gestational weight gain (GWG), gestational diabetes or hyperglycaemia and hypertensive disorders of pregnancy. Their links with offspring blood pressure (BP), adiposity and glucose–insulin traits are supported by systematic reviews and large cohorts, whereas evidence that a specific placental phenotype mediates the long-term association is more limited [27].

For maternal obesity, recent systematic reviews report broadly consistent associations with higher offspring BP, greater adiposity and less favourable lipid and insulin-resistance profiles, with some studies extending into adulthood [28,29]. The placenta may contribute to these associations by modifying the fetal metabolic, inflammatory and epigenetic environment. A meta-analysis of 10 cohorts comprising 2631 mother–infant pairs identified 27 placental CpG sites associated with maternal prepregnancy body mass index (BMI), with DNA methylation differences of up to 2.0% per 10-unit increase in prepregnancy BMI [30]. This supports a reproducible placental epigenetic correlate of maternal adiposity but does not show that these sites mediate offspring metabolic outcomes. In human terms, placenta and maternal obesity have been associated with alterations in placental morphology, vascularisation, nutrient transport, inflammatory signalling and endocrine function. Reported findings include altered expression of glucose and amino-acid transporters, lower vessel density and differences in IL-6, immune-cell infiltration, leptin and hCG production [31]. These changes occur in the context of insulin resistance, altered lipid metabolism and low-grade inflammation, which may influence placental nutrient sensing and fetal growth [31,32].

GWG provides pregnancy-specific information beyond prepregnancy BMI, as it reflects metabolic changes across gestation. In a systematic review and meta-analysis, higher GWG was associated with greater offspring fat mass, body-fat percentage and waist circumference, modest increases in systolic BP and triglycerides, and lower high-density lipoprotein (HDL) cholesterol across reported ages [33]. A systematic review of 49 studies identified epigenetic signatures associated with maternal BMI or GWG in maternal blood, placenta and offspring tissues; findings were more consistent for prepregnancy BMI than for GWG [34]. Individual human placental studies have nevertheless reported GWG-associated DNA methylation differences [35], while supraphysiological GWG has been associated with reduced fetoplacental vascular reactivity, downregulated endothelial nitric oxide synthase and altered adenosine transport [36]. These studies support the placenta as a plausible biological interface between GWG and fetal development, but they do not demonstrate that the measured placental changes mediate later cardiometabolic outcomes.

Pooled evidence indicates that gestational diabetes mellitus (GDM) and pregnancy hyperglycaemia are associated with higher offspring systolic BP, greater adiposity and higher glucose concentrations, whereas lipid findings are more heterogeneous [37,38]. A meta-analysis including more than 500,000 offspring found that the relative risk of overweight increased across age groups, although residual confounding by maternal adiposity and the postnatal environment remains possible [39]. Placental abnormalities most consistently reported in maternal diabetes include greater placental weight, villous immaturity and increased angiogenesis [40]. A GDM-specific meta-analysis found higher odds of delayed villous maturation (OR 6.37, 95% CI 3.28–12.37), while syncytial knotting and fibrin deposition were described in the narrative evidence; the small evidence base limits certainty [41]. Increased oxidative stress has also been reported across maternal, fetal and placental compartments, but substantial heterogeneity and possible publication bias require caution [42]. Longer-term follow-up strengthens the exposure–outcome association, with maternal glucose concentrations across the glycaemic spectrum associated with offspring adiposity and metabolic risk in late adolescence [43]. However, direct evidence that specific placental alterations mediate these longer-term cardiometabolic outcomes remains limited.

Hypertensive disorders of pregnancy, including preeclampsia, provide a complementary vascular exposure and are consistently associated with higher offspring BP [38,44]. A 2025 meta-analysis reported a mean offspring systolic BP difference of 2.44 mmHg (95% CI 2.03–2.85) after hypertensive disorders of pregnancy [45]. In adults, a later meta-analysis reported differences of 3.40 mmHg (95% CI 2.44–4.37) in systolic BP and 2.19 mmHg in diastolic BP, together with higher odds of hypertension (OR 1.50, 95% CI 1.18–1.91) [46]. Placental pathology is particularly prominent in preeclampsia, with villous and vascular lesions reported four to seven times more often than in normotensive pregnancies [47]. Meta-analytic evidence also indicates increased lipid peroxidation in blood and placenta and altered placental malondialdehyde, whereas reductions in antioxidant activity were more consistently observed in blood than in placental tissue [48]. These findings provide convergent evidence across the exposure–placenta and exposure–outcome links, but the relevant placental and adult-outcome data largely arise from separate studies. A nationwide cohort also associated maternal hypertensive disorders with offspring diabetes from childhood into early adulthood, with higher risk after more severe disease [49] (Table 1).

Evidence from interventions is limited but clinically informative, as it helps determine whether improving maternal metabolic conditions during pregnancy can modify early cardiovascular phenotypes in offspring. A systematic review of follow-up studies from lifestyle interventions in women with obesity concluded that such interventions may reduce markers of adverse cardiovascular remodelling in offspring, although outcomes are largely early-life proxies and follow-up into adulthood is scarce [50]. In the UPBEAT trial follow-up, an antenatal lifestyle intervention among women with obesity was associated with attenuation of cardiac remodelling patterns at age 3 years, providing an illustrative example that pregnancy exposures are modifiable and that offspring cardiovascular phenotypes may respond to maternal interventions [51]. These findings do not demonstrate prevention of adult disease. However, they suggest that pregnancy may be a period during which aspects of offspring cardiometabolic risk are modifiable.

Only a few human studies have assessed more than one stage of the proposed pathway within the same cohort. Thame et al., showed that maternal nutritional status predicted placental and fetal growth, while greater second-trimester placental volume was associated with lower childhood systolic blood pressure, supporting a potential placental pathway linking maternal nutrition with later cardiovascular development [52]. Ouyang et al. reported that placental weight statistically attenuated the associations of gestational diabetes, excessive GWG and prepregnancy BMI with childhood BMI by 52%, 25% and 17%, respectively [53]. In the Helsinki Birth Cohort, combinations of maternal body size and placental size or shape predicted coronary heart disease in adult men [54]. Norwegian data also linked higher placental weight relative to birth weight with cardiovascular mortality [55]. These studies provide more integrated human evidence than analyses of adjacent links alone, but placental size and weight are crude proxies, attenuation or prediction does not establish causal mediation, and studies combining molecular or histopathological placental phenotypes with adult outcomes remain scarce.

Overall, evidence from systematic reviews and large cohort studies supports associations between maternal obesity, hyperglycaemia, and hypertensive disorders and later offspring outcomes. Findings on placental abnormalities add biological plausibility to these associations, although longitudinal studies linking specific placental alterations with long-term, particularly adult, offspring outcomes remain limited.

## 4. Placental Neuroendocrine and Inflammatory Pathways Linking Prenatal Stress to Later Offspring Vulnerability

Prenatal psychosocial stress and maternal mental health conditions may influence offspring health through maternal neuroendocrine, immune and behavioural pathways that converge at the placenta. Epidemiological studies link prenatal stress and maternal psychological distress with a range of offspring outcomes, including altered stress responsivity, neurodevelopmental and psychiatric vulnerability, greater adiposity and, less consistently, cardiovascular and metabolic abnormalities. However, relatively few longitudinal studies have measured placental phenotypes together with long-term offspring outcomes.

Evidence based on severe acute stressors provides one of the clearest paths to understanding adult outcomes, since the timing of exposure can be linked to a specific event and the effects can be tracked over many years. In a nationwide Danish cohort including more than 2.6 million births followed for up to 40 years, prenatal exposure to maternal bereavement was associated with a modestly higher risk of CVD in offspring, although this association was weaker in within-family (sibling-matched) analyses. Overall, the hazard ratio (HR) for CVD was 1.13 (95% CI: 1.06–1.20); the estimate was 1.24 (95% CI: 1.11–1.38) for heart disease and 1.27 (95% CI: 1.01–1.60) for hypertension [56]. More recently, a binational Nordic registry study of 6.8 million individuals followed up to age 49 reported no overall association between prenatal exposure to maternal bereavement and ischaemic heart disease or stroke, although an increased risk of ischaemic heart disease was observed when the loss occurred in the third trimester [57]. Taken together, these findings suggest that any long-term cardiovascular effect of severe prenatal stress is likely to be modest and may depend on exposure timing, outcome definition and residual familial or social confounding.

The placenta provides a biologically plausible interface for these associations, particularly through regulation of fetal glucocorticoid exposure. Placental 11β-hydroxysteroid dehydrogenase type 2 (11β-HSD2), encoded by HSD11B2, converts active cortisol to inactive cortisone and thereby limits fetal glucocorticoid exposure. Human evidence has associated later-pregnancy anxiety with lower placental HSD11B2 expression [58], while a systematic review found modest, heterogeneous associations between prenatal psychological distress and placental 11β-HSD2, with the third trimester emerging most consistently [59]. Reduced placental glucocorticoid buffering could increase fetal cortisol exposure and alter HPA-axis development and postnatal cortisol reactivity, with some studies suggesting greater susceptibility in female offspring [60]. Maternal stress and depression have also been associated with placental inflammatory signalling, serotonin-related pathways, oxidative balance, nutrient transport and epigenetic regulation [61,62,63]. Together, these findings support a placental stress-response phenotype involving neuroendocrine, immune and metabolic pathways rather than a single molecular pathway.

Evidence for downstream metabolic consequences is also emerging. A human systematic review and meta-analysis associated prenatal stress with higher offspring BMI (standardised mean difference 0.27, 95% CI 0.19–0.35) and higher body-fat percentage, while evidence for BP, glucose regulation and lipids was sparse or inconsistent [64]. A broader scoping review incorporating human and experimental studies similarly suggested potential metabolic consequences, but differences in exposure definition, species, timing and outcome assessment limit direct translation [65]. Importantly, these offspring studies rarely include concurrent measures of placental glucocorticoid metabolism, inflammation or vascular function. Thus, the placental and later-outcome links largely derive from separate study populations, and direct longitudinal demonstration of placental mediation remains lacking (Table 2).

Pharmacological treatment during pregnancy further complicates causal interpretation. Antidepressant exposure is closely related to the underlying indication and severity of maternal psychiatric illness and may also correlate with smoking, diet, healthcare utilisation and other stress-related behaviours [66,67,68,69]. Pooled evidence has associated antidepressant use during pregnancy with pregnancy-induced hypertension, which may secondarily affect placental perfusion and increase the risk of preterm birth [70]. These pregnancy outcomes may therefore represent sentinel phenotypes of an altered intrauterine environment, but current evidence does not establish a pathway from antidepressant exposure through a specific placental lesion to long-term offspring disease.

**Table 2:** Prenatal stress and maternal mental health: evidence, offspring outcomes and proposed placental pathways.

Study	Evidence and Exposure	Outcome and Main Finding	Evidence Interpretation
O’Donnell et al. (2012) [58]	Human pregnancy cohort; maternal anxiety and placental glucocorticoid metabolism	Later-pregnancy anxiety was associated with lower placental HSD11B2 expression.	Exposure→placenta evidence; later offspring cardiometabolic outcomes were not measured.
Burgueño et al. (2020) [64]	Systematic review and meta-analysis of human studies; prenatal stress	Prenatal stress was associated with higher offspring BMI (SMD 0.27, 95% CI 0.19–0.35); other cardiometabolic outcomes were sparse or inconsistent.	Exposure→offspring evidence. Placental measures were generally absent.
Plana-Ripoll et al. (2020) [56]	Nationwide Danish cohort with sibling-matched analysis; maternal bereavement	Prenatal bereavement was associated with modestly higher offspring CVD risk into adulthood; estimates weakened in sibling analyses.	Exposure→adult outcome evidence; familial and social confounding remain important.
Eberle et al. (2021) [65]	Scoping review of human and experimental evidence; maternal stress	The review suggested possible effects on offspring adiposity and glucose metabolism, with marked heterogeneity across designs.	Biological plausibility and convergent evidence; not direct proof of placental mediation.
Tartour et al. (2024) [59]	Systematic review and meta-analysis; prenatal psychological distress	Distress showed modest, heterogeneous associations with lower placental 11β-HSD2, particularly in the third trimester.	Exposure→placenta evidence. Pooled effects were small and not linked directly to adult disease.
Hu et al. (2025) [70]	Systematic review and meta-analysis of 17 observational studies; antidepressant use during pregnancy	After accounting for depression, SNRI use remained associated with gestational hypertension or preeclampsia (OR 1.53, 95% CI 1.25–1.88), whereas associations for SSRIs and TCAs included the null.	Exposure→maternal pregnancy-complication evidence. No offspring outcome or direct placental phenotype was assessed; residual confounding by indication remains possible.

Abbreviations: CVD, cardiovascular disease; HSD11B2, hydroxysteroid 11-beta dehydrogenase 2.

Long-term associations between severe maternal stress and offspring outcomes are supported by cohort studies, while placental HSD11B2 findings provide a plausible human biological pathway. However, these elements have rarely been examined within the same longitudinal framework, and residual confounding related to familial, social, and treatment factors remains an important limitation.

## 5. Environmental and Dietary Exposures: Oxidative, Endocrine and Vascular Mechanisms

Pregnancy-period environmental and dietary exposures are shaped by geography, occupation, housing, and socioeconomic position, and they often co-occur with psychosocial stress and constrained access to health-promoting resources [71,72,73,74]. From an adult-health perspective, the central issue is whether pregnancy exposures contribute to cardiometabolic and metabolic risk that persists into late adolescence and early adulthood, independent of postnatal influences and shared family context. The most consistent evidence base relates to ambient air pollution, persistent pollutants such as per- and polyfluoroalkyl substances (PFAS), and selected endocrine-disrupting chemicals (EDCs), with diet and built-environment features functioning as modifiers and correlated determinants of risk across the life course [75,76,77]. Complementary biomonitoring investigations have demonstrated transplacental transfer of endocrine-disrupting compounds, including parabens and triclosan, confirming measurable fetal chemical burden during critical developmental windows of pregnancy [78]. Regulatory and dietary risk-assessment studies further demonstrate that endocrine-disrupting chemicals and persistent organic pollutants are detectable in infant formulas and baby foods, indicating continued exposure beyond the intrauterine period and supporting continuity of exposure across early developmental stages [79].

Ambient air pollution has drawn sustained attention because exposure can be estimated across large populations, and the resulting cardiovascular outcomes in adolescence and early adulthood are clinically meaningful and readily interpretable [80,81]. Across studies that consider both prenatal and postnatal periods, prenatal particulate matter 2.5 (PM2.5) has been associated with higher BP in childhood and adolescence, suggesting that in utero exposure represents one component of a broader, cumulative exposure history [82]. In a systematic review and meta-analysis, higher exposure to PM1, PM2.5, PM10, and nitrogen oxides (NO_x_) was associated with increased risk of overweight/obesity and elevated BMI in children and adolescents, with stronger associations observed at higher concentration levels and in Asian populations [83]. Direct adult-adjacent evidence is now available from a UK birth cohort that characterised air pollution exposure from pregnancy through age 18 and assessed cardiovascular markers at age 18, reporting that higher average PM2.5 and nitrogen dioxide (NO_2_) across this period were associated with higher peripheral and central diastolic BP. Although age 18 represents early adulthood rather than midlife disease, this design strengthens the adult-health narrative by demonstrating that exposure-associated differences in BP persist at the transition to adult care [84].

Biomarker studies during pregnancy help define pathways through which environmental exposures may contribute to later cardiometabolic vulnerability. Analyses of maternal biological matrices and amniotic fluid confirm fetal exposure to phthalates and other endocrine-disrupting chemicals during gestation [85]. In trimester-specific analyses, prenatal air pollution was associated with inflammatory, cardiovascular and metabolic biomarkers in mothers and newborns, supporting maternal inflammatory and endothelial pathways that may affect placental perfusion and fetal vascular development [86]. Prenatal PM2.5 exposure has also been associated with greater arterial stiffness in childhood, measured by pulse-wave velocity [87]. Arterial stiffness is relevant to later cardiovascular risk at the population level, but these studies do not demonstrate that a measured placental abnormality mediated the association. Studies of adiposity likewise frequently combine prenatal and postnatal windows, limiting isolation of a fetal-life effect.

Persistent pollutants are important in developmental origins research because exposure often begins in utero, and several compounds can disrupt endocrine and metabolic regulation during critical developmental windows [88,89]. For PFAS, a systematic review and meta-analysis reported associations between prenatal and early-life PFAS exposure and overweight/obesity in children older than 3 years, with effects varying by specific compound and sex. These findings show the importance of compound-specific interpretation rather than a single pooled direction of effect [90]. More detailed cardiometabolic phenotyping is available from the Canadian MIREC cohort, in which second-trimester maternal plasma PFAS concentrations were examined in relation to offspring BP, adiposity, lipids, and insulin resistance at ages 7–9 years. The analysis incorporated both mixture modelling and single-exposure approaches, with the direction and magnitude of associations varying by specific compound and cardiometabolic outcome [91]. Adult outcomes after pregnancy and PFAS exposure remain limited, but these childhood profiles are clinically relevant, as they are well-established precursors of adult cardiometabolic morbidity and can help prioritise longer-term follow-up.

Evidence for EDCs such as bisphenols and phthalates is heterogeneous, reflecting differences in biomarkers, timing, mixtures, and outcome definitions, but several reviews support pregnancy exposure as a contributor to later adiposity-related phenotypes. A systematic review focused on prenatal bisphenol A (BPA) concluded that prenatal BPA exposure is associated with offspring obesity and related anthropometric outcomes in multiple cohorts, with evidence of sex- and age-related differences across studies [92]. For phthalates, a systematic review of metabolic syndrome components reported that prenatal exposure to several phthalates is associated with markers of adiposity in offspring, whereas postnatal exposure appeared more consistently linked to glycaemic traits, further supporting the importance of considering the timing of exposure [93]. A meta-analysis reported a significant inverse association between prenatal di(2-ethylhexyl) phthalate (DEHP) exposure and offspring BMI z-score, while no association was observed with body fat percentage. This pattern is compatible with prenatal phthalate exposure influencing growth patterns rather than directly promoting adiposity, as associations may differ across outcomes and should not be interpreted as uniformly supporting obesity-related hypotheses [94]. From an adult-health perspective, the principal limitation is the relative scarcity of adult follow-up; nonetheless, childhood adiposity and BP are trajectories that often persist into adulthood and remain relevant endpoints within a life-course argument.

Maternal diet and the built environment provide a complementary aspect because they shape fetal nutrient supply and maternal metabolic status, and they are correlated with chemical exposures and social determinants across pregnancy and later life. Across three US prospective cohorts, the Nurses’ Health Study II (NHSII) and the Growing Up Today Study (GUTS I and II) in the United States, higher maternal consumption of ultra-processed foods was associated with higher risk of offspring overweight or obesity through adolescence. However, because the exposure period was not limited to pregnancy and analyses around the peripregnancy period were limited, these findings are better viewed as reflecting broader family dietary patterns and the link between pregnancy diet and the child’s later food environment [95]. For the built environment, a large meta-analysis reported that greater greenspace exposure is associated with lower odds of hypertension, obesity, and diabetes, but pregnancy-specific exposure definitions were not consistent, and the evidence should be positioned as contextual support rather than direct proof of effects limited to pregnancy [96]. Overall, the environmental literature concerning fetal life supports attention to clustered exposures: air pollution, persistent chemicals, and diet frequently co-occur with socioeconomic adversity, and risk reduction is likely to require integrated antenatal and public health approaches rather than single-factor messaging (Table 3).

Among environmental exposures, air pollution has the most consistent support from longitudinal studies and biomarker-based investigations. Evidence for PFAS, bisphenols, and phthalates is more heterogeneous and varies according to the specific compound, timing of exposure, offspring sex, and outcome assessed. Longitudinal studies directly linking a defined environmental exposure with a specific placental phenotype and subsequent adult disease remain scarce, and most proposed placental pathways therefore rely on converging rather than direct evidence.

## 6. Preterm Birth and Fetal Growth Restriction as Clinically Observable Sentinel Phenotypes

Preterm birth and FGR are clinically observable sentinel phenotypes that may reflect several adverse maternal–placental–fetal pathways and are themselves associated with long-term cardiometabolic risk [97,98,99,100,101]. They should not be treated as simple intermediates. Preterm birth may follow infection and inflammation, cervical dysfunction, membrane rupture, maternal vascular disease, placental pathology, fetal disease or medically indicated delivery. FGR is frequently associated with placental insufficiency but may also arise from fetal, maternal or environmental causes. Neither phenotype is specific to placental dysfunction, and neither is an obligatory mediator between a prenatal exposure and adult disease.

Large register-based cohorts provide the strongest evidence linking preterm birth to adult outcomes, with risks varying systematically by gestational age. In a national Swedish cohort study, individuals born preterm had increased risks of hypertension into adulthood, with the highest relative risks among those born at the lowest gestational ages and with persistence into the fourth decade of life [102]. Using the same national data infrastructure, preterm birth has also been linked to adult heart failure, again showing a graded relationship by gestational age that extends beyond childhood cardiovascular traits to a clinically significant adult outcome [103]. Adult cerebrovascular outcomes have also been examined. In a national cohort using a co-sibling design, adults born preterm had higher risks of stroke; although within-family estimates were attenuated, they remained above the null, suggesting that pregnancy-related factors contribute to risk beyond shared familial influences [104].

These register-based findings are further supported by clinician-focused reviews that link epidemiological evidence to alterations in cardiovascular structure and physiology among adults born preterm. Preterm birth has been associated with higher BP, altered vascular development, and distinct cardiac structure and function in adulthood, providing a coherent clinical narrative linking early delivery to later cardiovascular vulnerability [105]. An additional review summarised adult echocardiographic and functional findings, including smaller cardiac chamber size and altered ventricular function, and positioned these phenotypes as clinically relevant substrates for heart failure risk later in life [106]. Together, these findings support the view that disruption of late-gestation cardiovascular development, combined with altered postnatal adaptation in the preterm circulation, may result in persistent differences in haemodynamics and myocardial structure that remain evident in adulthood.

Adult metabolic and renal outcomes further reinforce the understanding that the long-term consequences of prematurity are multisystemic, extending well beyond the cardiovascular domain [107,108,109]. A Swedish cohort study reported higher diabetes risk in young adults born preterm, with evidence of a gestational-age gradient, suggesting that early delivery may contribute to long-term metabolic vulnerability that may be evident by early adulthood [110]. In another national cohort, preterm birth was associated with an increased risk of chronic kidney disease from childhood through mid-adulthood, supporting the link between reduced nephron endowment, altered renal development and later hypertension and cardiometabolic risk [111]. These conditions develop over decades and are best understood as the cumulative result of long-term alterations in BP regulation, renal reserve, and metabolic control, rather than as isolated late-onset events.

Adult morbidity after preterm birth may also present as clustered disease burden. A population-based cohort study in Finland examined chronic disease multimorbidity in adolescence and early adulthood and reported higher risks among those born preterm, extending the evidence from single-outcome analyses to a clinically recognisable pattern of multi-diagnosis vulnerability early in adulthood [112]. Mental health and psychosocial functioning are similarly relevant for adult care, both because psychological morbidity contributes directly to adult wellbeing and because it can influence healthcare engagement and cardiometabolic risk behaviours [113,114]. In a cohort followed to age 35 years, adults born preterm showed differences in psychological and physical health indicators, supporting the inclusion of mental health outcomes within a broader adult-health profile of prematurity [114].

Although the evidence table is weighted towards prematurity, FGR and small-for-gestational-age (SGA) birth provide a parallel clinical signal. FGR denotes failure to achieve growth potential and is not interchangeable with SGA, which includes constitutionally small infants. When FGR arises with placental malperfusion, chronic fetal undernutrition or hypoxia may alter vascular, renal and metabolic development; however, placental insufficiency should be documented rather than inferred from size alone [98,101,113,115]. FGR and preterm birth frequently overlap, and the combined phenotype may identify greater later vulnerability, but heterogeneity in aetiology and classification limits simple causal interpretation.

From a preventive medicine perspective, preterm birth and FGR can serve as pragmatic markers for earlier BP, metabolic and renal assessment in young adults. Register-based associations with hypertension, heart failure, stroke, diabetes and chronic kidney disease support recording gestational age and fetal growth history, particularly in early-onset hypertension or multimorbidity. Evidence strength is highest for the association between gestational age and later clinical outcomes. It is weaker for any single upstream placental mechanism, because the same birth phenotype can arise through different pathways, and long-term studies rarely include detailed placental histopathology or molecular phenotyping (Table 4).

## 7. Molecular Domains of Biological Embedding: Epigenetic Signatures, Telomere Biology and Mitochondrial Function

Placental and offspring epigenetic signatures, telomere biology and mitochondrial function provide measurable molecular readouts of prenatal exposure at birth or during childhood [116,117,118]. These domains capture different biological processes and should not be treated as interchangeable measures of ageing. Telomere length reflects chromosome-end maintenance and accumulated cellular stress; mitochondrial DNA (mtDNA) abundance or integrity reflects aspects of mitochondrial biology; and DNA methylation signatures capture tissue-, cell- and algorithm-specific epigenetic variation [119,120]. Maternal–neonatal correlations in telomere length support an intrauterine and inherited component, but none of these measures is a clinical outcome or a validated individual predictor of adult cardiometabolic disease [121].

Telomere length (TL) is the most frequently studied marker in this literature. Maternal stress, smoking, air pollution and heavy-metal exposure during pregnancy have been associated with shorter or altered newborn TL, although effect direction and magnitude vary by exposure window, tissue and assay [122,123,124,125,126]. Pooled evidence supports shorter newborn TL after maternal smoking and an overall inverse association with prenatal metal exposure, with more consistent findings for cadmium and later-pregnancy exposure [124,125]. These associations show that prenatal exposures can coincide with early differences in telomere biology; they do not establish that neonatal TL mediates adult disease. In adults, shorter leukocyte TL has been associated with a higher risk of frailty independently of chronological age and other established risk factors [127], but this observation does not establish that neonatal TL predicts later frailty.

Mitochondrial measures form a related but distinct domain. Prenatal air-pollution studies have examined TL together with mtDNA content or related indices and reported exposure-associated differences at birth [123]. Oxidative and inflammatory stress can damage telomeric DNA and alter mitochondrial function, while mitochondrial reactive oxygen species may further affect telomere maintenance [128,129,130]. The placenta regulates fetal exposure to inflammatory and oxidative mediators and is frequently sampled in these studies [123,131]. However, mtDNA copy number is not equivalent to mitochondrial function, and cross-study comparison is limited by tissue selection, cell composition, extraction methods and adjustment for parental or maternal characteristics.

Epigenetic signatures provide a third domain. DNA methylation–based age estimates combine selected cytosine–phosphate–guanine sites using algorithms trained for particular ages and tissues [132,133]. Epigenetic age acceleration denotes an estimate higher than expected for chronological age, but paediatric clocks differ in their CpG content, training samples and performance across tissues. In prenatal research, these measures indicate exposure-associated methylation patterns; they should not be interpreted as literal acceleration of whole-organism ageing or as evidence of causal mediation.

In a Canadian prospective cohort, second-trimester bisphenol and phthalate biomarkers showed sex-specific associations with infant epigenetic age acceleration in buccal cells at 3 months [134]. Prenatal and early-life air-pollutant exposure was also associated with epigenetic age estimates at 6 years when several algorithms were applied [135]. These observations extend molecular associations beyond birth, but algorithm choice, tissue source and cell composition remain major sources of variability. Their prognostic relevance for adult cardiometabolic disease has not been established.

Metabolomic, proteomic, lipidomic, inflammatory and immune signatures represent additional molecular dimensions of placental dysfunction, but their links with later offspring health remain comparatively underexplored. A systematic review of 32 human placental metabolomics studies identified recurring perturbations in amino-acid, lipid and energy-related pathways across pregnancy complications, but the outcomes were confined largely to pregnancy and birth [136]. A systematic review of maternal and placental lipidomics likewise identified exposure- and complication-related lipid signatures associated mainly with pregnancy outcomes and fetal growth [137]. Integrative placental omics in diabetes has highlighted recurrent RNA and protein pathways, although most evidence still ends at delivery and requires external replication [138]. A birth cohort of 1803 mother–child pairs extended the placental link beyond birth: placental inflammatory and oxidative-stress mRNA markers were associated with fasting glucose, insulin and prediabetes risk at 5–6 years [139]. This is comparatively integrated placenta-to-child evidence, but it does not include a single defined maternal exposure and does not establish adult disease. Standardisation, tissue and cell-type specificity, multiple-testing correction, experimental confirmation and independent replication remain important challenges (Table 5).

## 8. Discussion

The evidence reviewed here supports a maternal–placental–fetal–life-course framework rather than a uniform pathway. Maternal metabolic, vascular, psychosocial and environmental exposures may alter fetal development through placental and non-placental pathways, while postnatal conditions influence whether early differences persist, diminish or become clinically apparent. The placenta is a central integrative organ because it senses and responds to maternal nutrients, hormones, inflammatory mediators and environmental agents through vascular, transport, endocrine and molecular functions [140,141,142]. Its centrality, however, should not be conflated with universal or proven mediation.

Maternal metabolic and vascular exposures during pregnancy, including obesity, excessive GWG, gestational diabetes and hypertensive disorders, may alter placental perfusion, nutrient transport, endocrine and inflammatory signalling, oxidative balance and vascular function, with potential consequences for fetal growth and later cardiometabolic health. Across systematic reviews and large population-based cohorts, these exposures are associated with higher offspring BP, adiposity and adverse glucose–insulin traits extending into adolescence and adulthood. Clinically recognisable sentinel phenotypes, including preterm birth and FGR, are associated with later hypertension, ischaemic heart disease, heart failure, stroke, diabetes and chronic kidney disease. These associations support life-course surveillance but do not identify a single placental pathway or imply deterministic risk.

At the same time, pregnancy exposures are socially patterned and often cluster with postnatal environmental and familial factors [60,123]. Separating intrauterine influences from shared genetic, behavioural, and socioeconomic factors remains challenging, even in sibling comparisons and other family-based designs [56,60]. Nevertheless, pregnancy offers a distinctive opportunity for prevention because it is routinely monitored in clinical care, occurs within a defined time frame, and involves exposures that are often modifiable. The most appropriate interpretation of the evidence is not deterministic, in the sense that pregnancy alone determines adult disease risk, but stratificational, whereby pregnancy history helps identify individuals or groups who may benefit from earlier surveillance and targeted preventive strategies [32,113].

Biological embedding is supported by molecular associations, but the markers should remain conceptually distinct. Prenatal smoking, air pollution and metal exposure have been associated with neonatal TL or mitochondrial indices, while selected chemical exposures have been associated with placental or childhood DNA methylation signatures. These measures may capture exposure, susceptibility or response. They are not interchangeable indicators of ageing, and current evidence does not establish that they mediate adult disease or provide reliable individual risk prediction.

Across exposure domains, evidence is strongest when long-term offspring associations converge with measured placental abnormalities, but only a minority of studies assess these components within the same cohort. Heterogeneity in exposure timing, placental tissue and phenotype, assay platform, outcome definition, covariate adjustment and follow-up limits cross-study comparison. Small molecular studies and selective reporting may inflate apparently coherent signals. Many placental pathways should thus be regarded as plausible or convergent rather than established mediators of adult disease.

Clinically, the evidence supports incorporating pregnancy history into adolescent and early adult cardiometabolic risk assessment. Maternal obesity, excessive gestational weight gain, gestational diabetes, and hypertensive disorders identify groups with a higher probability of later obesity, higher BP, and less favourable metabolic profiles, even when effect sizes are modest for individuals. Because these exposures cluster with social determinants and postnatal lifestyle factors, the most constructive application is targeted prevention rather than deterministic labelling, including anticipatory guidance, support for healthy growth trajectories, and early BP and metabolic screening where indicated. Documentation and structured communication of antenatal metabolic and vascular complications at discharge, followed by integration into primary care records, could improve continuity and align reproductive history with adult prevention strategies [113,115].

### Limitations

Several limitations restrict the conclusions. Exposure timing, biological matrices, placental sampling, histopathological definitions and methods for mixtures differ widely between studies. Outcomes range from birth biomarkers to adult clinical events, and molecular estimates depend on tissue, cell composition, assay platform and algorithm. Residual genetic, socioeconomic, behavioural and familial confounding remains possible, even in sibling or family-based designs, while differential loss to follow-up may select healthier participants. Many molecular studies are small and imprecise, increasing susceptibility to selective reporting and publication bias. As a structured narrative review, this article did not use a single deduplicated screening set, formal study-level risk-of-bias scoring or certainty grading; evidence-strength categories were interpretive rather than equivalent to GRADE or a systematic-review framework.

Methodological priorities include harmonised placental histopathology and molecular phenotyping, repeated exposure and offspring assessment, adequate control of tissue and cell composition, and transparent reporting of quality control, normalisation and analytic choices. Long-term follow-up is needed to determine whether molecular differences observed at birth persist and align with vascular, metabolic or renal phenotypes. Current studies support measurable biological embedding during fetal life [73,80,115], but they do not yet provide validated causal or prognostic markers for adult disease.

## 9. Conclusions and Future Directions

Current human evidence supports pregnancy as a sensitive developmental window in which maternal exposures, placental responses, fetal adaptation and postnatal conditions jointly shape later cardiometabolic susceptibility. Maternal metabolic and vascular disorders show the most consistent long-term associations, while environmental, psychosocial and molecular evidence is more heterogeneous. Preterm birth and FGR are clinically useful sentinel phenotypes but are neither specific to placental dysfunction nor obligatory mediators. The principal knowledge gap is the scarcity of longitudinal human studies measuring a defined maternal exposure, a specific placental phenotype, fetal organ development, childhood cardiometabolic trajectories and adult clinical outcomes within the same design.

Future studies should combine harmonised placental histopathology with epigenomic, transcriptomic, proteomic, metabolomic, lipidomic, inflammatory and immune profiling, followed by repeated assessment of offspring vascular and metabolic phenotypes into adulthood. Interpretable machine-learning models may help identify cross-omic signatures but require prespecified external validation and cannot replace causal design. Pragmatic antenatal and preconception trials should assess whether modifying maternal metabolic, vascular or environmental exposures changes placental function, fetal organ development and later cardiometabolic trajectories.

## Figures and Tables

**Table 1:** Maternal metabolic and vascular health: exposure, placental pathway and offspring cardiometabolic outcome.

Study	Evidence and Exposure	Outcome and Main Finding	Evidence Interpretation
Pardo et al. (2015) [36]	Human fetoplacental vascular study; supraphysiological GWG	Higher GWG was associated with reduced fetoplacental vascular reactivity, downregulated eNOS activity and altered adenosine transport.	Exposure→placenta evidence. Later offspring outcomes were not assessed.
Falco et al. (2017) [47]	Systematic review; preeclampsia and placental histopathology	Villous and vascular lesions were reported four to seven times more often in preeclamptic than normotensive pregnancies.	Exposure→placenta evidence. Adult offspring outcomes were not assessed.
Pathirana et al. (2020) [37]	Systematic review of 59 studies and meta-analysis of 24 studies; offspring exposed to maternal GDM	GDM-exposed offspring had higher SBP (MD +1.75 mmHg, 95% CI 0.57–2.94), BMI z-score (MD + 0.11, 95% CI 0.02–0.20) and glucose concentrations (SMD 0.43, 95% CI 0.08–0.77).	Exposure→offspring evidence. Differences in cardiometabolic risk factors were modest, and placental phenotypes or mediation pathways were not assessed.
Fernandez-Jimenez et al. (2022) [30]	Meta-analysis of 10 cohorts (2631 mother–infant pairs); maternal prepregnancy BMI	Twenty-seven placental CpG sites were associated with prepregnancy BMI; DNA methylation differences reached 2.0% per 10-unit BMI increase.	Exposure→placenta evidence. Reproducible epigenetic association, but no long-term offspring outcome or mediation analysis.
Gao et al. (2022) [39]	Meta-analysis of 49 cohorts (>500,000 offspring); maternal GDM	The relative risk of offspring overweight increased across age groups and remained elevated in adulthood.	Exposure→offspring evidence. Residual confounding by maternal adiposity and the postnatal environment remains possible.
Arcot et al. (2025) [41]	Systematic review and meta-analysis; GDM and placental lesions	GDM was associated with delayed villous maturation (OR 6.37, 95% CI 3.28–12.37); syncytial knotting and fibrin were reported narratively.	Exposure→placenta evidence. Small evidence base; no long-term outcome link.
Deng et al. (2025) [43]	Prospective birth cohort; maternal OGTT; offspring followed to 18 years	Higher maternal glucose across the glycaemic spectrum was associated with adverse glucose indices and overweight/obesity at 18 years.	Exposure→offspring evidence. No placental phenotype was measured.
Wen et al. (2025) [33]	Systematic review and meta-analysis; gestational weight gain	Higher GWG was associated with greater offspring adiposity, modestly higher SBP and triglycerides, and lower HDL-C.	Exposure→offspring evidence. Placental measurements were not required.
Saji et al. (2026) [46]	Systematic review and meta-analysis; adult offspring exposed to hypertensive disorders of pregnancy	Adult offspring had higher SBP (+3.40 mmHg) and DBP (+2.19 mmHg) and higher odds of hypertension (OR 1.50).	Exposure→adult outcome evidence. Placental phenotypes were not integrated.

Abbreviations: BMI, body mass index; CI, confidence interval; CpG, cytosine–phosphate–guanine; DBP, diastolic blood pressure; DNA, deoxyribonucleic acid; eNOS, endothelial nitric oxide synthase; GDM, gestational diabetes mellitus; GWG, gestational weight gain; HDL-C, high-density lipoprotein cholesterol; OGTT, oral glucose tolerance test; OR, odds ratio; SBP, systolic blood pressure.

**Table 3:** Environmental and dietary exposures during pregnancy: evidence, offspring outcomes and proposed placental pathways.

Study	Evidence and Exposure	Outcome and Main Finding	Proposed Pathophysiological Link
Lee et al. (2022) [94]	Systematic review and meta-analysis; prenatal phthalates, including DEHP	Prenatal DEHP was associated with lower child BMI z-score; body fat percentage findings were not significant	The evidence does not support a uniform obesogenic effect. Phthalates may influence growth patterning rather than directly increasing adiposity.
Wang et al. (2022) [95]	Prospective cohorts; maternal ultra-processed food intake, not restricted to pregnancy	Higher maternal ultra-processed food intake was associated with higher offspring overweight/obesity risk through adolescence	This likely reflects both maternal diet and the later family food environment. A pregnancy-specific fetal pathway cannot be separated clearly from postnatal dietary continuity.
Frigerio et al. (2023) [90]	Systematic review with meta-analyses; PFAS exposure including pregnancy-period exposure	PFAS associations with childhood overweight/obesity varied by compound and sex, with an overall signal for adiposity outcomes	PFAS may contribute to adiposity risk through endocrine and lipid-metabolic pathways, but the direction and size of effects depend on compound and sex.
Liu et al. (2023) [82]	Systematic review and meta-analysis; prenatal PM2.5 within broader pre/postnatal evidence	Prenatal PM2.5 was associated with higher child/adolescent BP and hypertension risk	Prenatal particulate exposure may affect BP regulation through placental inflammation, oxidative stress and altered fetal vascular development.
Guo et al. (2024) [92]	Systematic review of human cohort studies; prenatal BPA exposure	Prenatal BPA exposure was generally associated with offspring obesity or adverse anthropometric outcomes; sex- and age-related differences were reported	BPA may affect adiposity-related development through endocrine signalling, adipogenesis and sex-specific metabolic effects.
Ji et al. (2024) [86]	MADRES pregnancy cohort; PM2.5, PM10, NO_2_, O_3_ and traffic-related NO_x_; maternal and cord-blood biomarker panels	Prenatal air pollutants were associated with distinct maternal and cord-blood biomarker patterns involving inflammatory, cardiovascular and metabolic pathways	These biomarker patterns support a pathway in which prenatal air pollution affects fetal exposure through maternal inflammation, endothelial stress and altered cord-blood metabolic signalling.
Perez-Diaz et al. (2024) [93]	Systematic review; phthalate exposure, with prenatal findings summarised separately where available	Prenatal phthalates were linked with adiposity markers, whereas postnatal exposure was more consistently related to glucose traits	Phthalates may influence metabolic development through endocrine disruption and altered adipose or glucose regulation. Timing of exposure appears important.
Sharifi et al. (2024) [96]	Systematic review and meta-analysis; greenspace exposure, not consistently pregnancy-specific	Greater greenspace exposure was linked with lower odds of hypertension, obesity and diabetes	Greenspace may reflect lower pollution, less heat stress, more physical activity and broader socioeconomic context. This is useful contextual evidence, but not direct proof of a pregnancy-specific mechanism.
Zheng et al. (2024) [83]	Systematic review and meta-analysis; ambient air pollution, including prenatal and postnatal windows	Higher exposure was associated with higher odds of overweight/obesity in children and adolescents	Air pollution may influence adiposity through inflammation, oxidative stress and metabolic regulation, but many studies combine prenatal and postnatal exposure windows.
Fossa et al. (2025) [91]	Prospective Canadian cohort; second-trimester maternal plasma PFAS, assessed as single compounds and mixtures	Associations with BP, adiposity, lipids and insulin resistance at 7–9 years differed by compound and outcome	PFAS may affect metabolic and vascular traits through endocrine, lipid-metabolic and inflammatory pathways. Compound-specific effects are important and should not be collapsed into a single direction.
Goncalves Soares et al. (2025) [84]	UK birth cohort; air pollution assessed from pregnancy through adolescence; follow-up at 18 years	Higher average PM2.5 and NO_2_ exposure was linked with higher peripheral and central diastolic BP at 18 years	Long-term exposure to particulate and traffic-related pollution may affect vascular development and later BP through endothelial stress and inflammatory pathways. The effect cannot be assigned to pregnancy alone.
Renaers et al. (2025) [87]	Birth cohort analysis; prenatal PM2.5 exposure	Higher prenatal PM2.5 was associated with higher childhood pulse wave velocity	Prenatal particulate exposure may contribute to early vascular stiffening, possibly through oxidative stress, endothelial dysfunction and altered arterial development.

Abbreviations: BMI, body mass index; BP, blood pressure; BPA, bisphenol A; DEHP, di(2-ethylhexyl) phthalate; NO_2_, nitrogen dioxide; NO_x_, nitrogen oxides; O_3_, ozone; PFAS, per- and polyfluoroalkyl substances; PM, particulate matter; PM2.5, particulate matter ≤ 2.5 μm; PM10, particulate matter ≤ 10 μm.

**Table 4:** Preterm birth as a clinically observable sentinel phenotype: adult outcomes and proposed pathways.

Study	Evidence and Sentinel Phenotype	Adult Outcome and Main Finding	Evidence Interpretation
Crump et al. (2011) [110]	Swedish population-based cohort; gestational age categories	Preterm birth was associated with higher diabetes risk in young adulthood.	Altered pancreatic, adipose and metabolic development may contribute to later glucose dysregulation.
Crump et al. (2019) [111]	Register-based cohort; preterm birth	Preterm birth was associated with higher risk of chronic kidney disease.	Early delivery may reduce nephron endowment and renal reserve.
Crump et al. (2020) [102]	Population-based cohort; gestational age at birth	Lower gestational age was associated with higher hypertension risk in adulthood.	Reduced vascular and renal maturation may contribute to higher adult BP.
Lewandowski et al. (2020) [105]	Review of adult cardiovascular phenotypes after preterm birth	Adults born preterm showed higher BP, altered cardiac structure and higher CVD risk.	Preterm birth may leave a distinct cardiovascular phenotype.
Crump et al. (2021) [103]	Population-based cohort; gestational age at birth	Lower gestational age was associated with higher heart-failure risk up to early adulthood.	Disrupted myocardial development and altered postnatal loading may increase susceptibility.
Crump et al. (2021) [104]	Register-based cohort; preterm birth	Preterm birth was associated with higher stroke risk in adulthood, with a gestational-age gradient.	Altered vascular development, higher BP and endothelial dysfunction may contribute.
Singer et al. (2021) [113]	Narrative review; preterm birth	Adult morbidity extended across cardiovascular, metabolic and neuropsychological domains.	The later phenotype is multisystem rather than attributable to one pathway.
Greer et al. (2022) [106]	Review of adult cardiac imaging and functional findings after preterm birth	Adults born preterm showed consistent differences in left-ventricular structure and function.	Interruption of late-gestation cardiac development may contribute to later cardiovascular vulnerability.
Heikkilä et al. (2022) [112]	Population-based cohort; preterm birth	Individuals born preterm had higher overall CVD risk, including outcomes extending into adulthood.	Preterm birth may mark persistent vascular and metabolic vulnerability.
D’Agata et al. (2025) [114]	Cohort follow-up; adults born preterm	Adults born preterm showed a higher burden of selected psychological outcomes and differences in physical health at 35 years.	Preterm birth may reflect early disruption of neurodevelopment, stress regulation and somatic growth.

Abbreviations: BP, blood pressure; CVD, cardiovascular disease.

**Table 5:** Molecular domains linked to exposures during pregnancy.

Study	Evidence and Exposure	Molecular Domain	Main Finding
England-Mason et al. (2024) [134]	Prospective cohort; prenatal bisphenols and phthalates	Infant epigenetic age acceleration	Associations at 3 months differed by chemical, sex and metabolite.
Lee et al. (2024) [135]	Birth cohort; prenatal and early-life air pollutants	Epigenetic age acceleration at 6 years	Several prenatal and early-life pollutants were associated with epigenetic age estimates; algorithm choice remained important.
Mishra et al. (2024) [123]	Systematic review; prenatal ambient air pollution	Telomere length and/or mtDNA at birth	Prenatal air pollution was associated with altered telomere length and/or mtDNA, with variable direction and sensitive windows.
Wai et al. (2024) [124]	Systematic review and meta-analysis; prenatal metal exposure	Newborn telomere length	Prenatal metal exposure was associated overall with shorter newborn telomere length; cadmium and later-pregnancy windows were more consistent.
Zhou et al. (2024) [139]	Birth cohort of 1803 mother–child pairs	Placental inflammatory and oxidative-stress mRNA	Placental markers were associated with fasting glucose, insulin and prediabetes risk at 5–6 years; adulthood was not assessed.
Cohen et al. (2025) [131]	Narrative review; chemical, psychosocial and metabolic prenatal exposures	Placental DNA methylation	The review emphasised fetal sex, cell composition, mediation analysis, multi-omics and cautious interpretation.
Kalapouti et al. (2025) [137]	Systematic review; maternal and placental lipidomics	Lipidomic signatures	Lipid profiles were linked mainly with pregnancy outcomes and fetal growth rather than later disease.
Fakonti et al. (2026) [138]	Integrative review of placental omics in diabetes	Transcriptomic and proteomic pathways	Recurrent RNA and protein pathways were identified, but most evidence ended at delivery.
Mercer and Cahill (2026) [136]	Systematic review of 32 human placental metabolomics studies	Placental metabolome	Recurring amino-acid, lipid and energy-related pathways were reported across pregnancy complications; long-term offspring outcomes were absent.
Moshfeghinia et al. (2026) [125]	Systematic review and meta-analysis; maternal smoking during pregnancy	Newborn telomere length	Maternal smoking during pregnancy was associated with shorter newborn telomere length.

Abbreviation: mtDNA, mitochondrial DNA.

## Data Availability

No new data were created or analyzed in this study. Data sharing is not applicable.

## Abbreviations

The following abbreviations are used in this manuscript:

11β-HSD2	11β-hydroxysteroid dehydrogenase type 2
BMI	Body mass index
BP	Blood pressure
BPA	Bisphenol A
CI	Confidence interval
CKD	Chronic kidney disease
CpG	Cytosine–phosphate–guanine (dinucleotide site)
CVD	Cardiovascular disease
DBP	Diastolic blood pressure
DEHP	Di(2-ethylhexyl) phthalate
DNAm	DNA methylation
DOHaD	Developmental origins of health and disease
EAA	Epigenetic age acceleration
EDCs	Endocrine-disrupting chemicals
fMRI	Functional magnetic resonance imaging
FGR	Fetal growth restriction
GDM	Gestational diabetes mellitus
GLUT1	Glucose transporter 1
GWG	Gestational weight gain
hCG	Human chorionic gonadotropin
HDL-C	High-density lipoprotein cholesterol
HPA axis	Hypothalamic–pituitary–adrenal axis
HR	Hazard ratio
IHD	Ischaemic heart disease
IL-6	Interleukin-6
LV	Left ventricle/left ventricular
mtDNA	Mitochondrial DNA
NCDs	Non-communicable diseases
NO_2_	Nitrogen dioxide
NO_x_	Nitrogen oxides
O_3_	Ozone
OGTT	Oral glucose tolerance test
OR	Odds ratio
PFAS	Per- and polyfluoroalkyl substances
PM	Particulate matter
PM2.5	Particulate matter ≤2.5 μm (aerodynamic diameter)
PM10	Particulate matter ≤10 μm (aerodynamic diameter)
PWV	Pulse wave velocity
RCT	Randomised controlled trial
SBP	Systolic blood pressure
SGA	Small for gestational age
SNAT1–2	Sodium-coupled neutral amino acid transporter 1–2
TG	Triglycerides
TL	Telomere length
UPBEAT	UK Pregnancies Better Eating and Activity Trial
